# A Novel Partial Sequence Alignment Tool for Finding Large Deletions

**DOI:** 10.1100/2012/694813

**Published:** 2012-04-01

**Authors:** Taner Aruk, Duran Ustek, Olcay Kursun

**Affiliations:** ^1^Scientific and Technological Research Council of Turkey (TUBITAK), 41470 Kocaeli, Turkey; ^2^Genetics Department, Institute for Experimental Medicine, Istanbul University, 34093 Istanbul, Turkey; ^3^Computer Engineering Department, Istanbul University, 34320 Istanbul, Turkey

## Abstract

Finding large deletions in genome sequences has become increasingly more useful in bioinformatics, such as in clinical research and diagnosis. Although there are a number of publically available next generation sequencing mapping and sequence alignment programs, these software packages do not correctly align fragments containing deletions larger than one kb. We present a fast alignment software package, BinaryPartialAlign, that can be used by wet lab scientists to find long structural variations in their experiments. For BinaryPartialAlign, we make use of the Smith-Waterman (SW) algorithm with a binary-search-based approach for alignment with large gaps that we called partial alignment. BinaryPartialAlign implementation is compared with other straight-forward applications of SW. Simulation results on mtDNA fragments demonstrate the effectiveness (runtime and accuracy) of the proposed method.

## 1. Introduction

In bioinformatics, sequence alignment is a way of arranging the sequences of DNA, RNA, or protein to identify regions of similarity that may be a consequence of functional, structural, or evolutionary relationships between the sequences [1]. Next Generation sequencing (NGS) technology produces terabytes of sequencing data in an inexpensive way [2]. However, among these sequences, most NGS software such as Path, UGENE, JAligner, SSEARCH, Water, and others based on the well-known Smith-Waterman algorithm (SW) [3–5] is designed to align sequences with small gaps and may not be suitable when large deletions are present. Finding such large deletions (and the related counterpart, finding large insertions) has become increasingly more useful in bioinformatics [6, 7]. It has been long known that chromosomal deletions can lead to developmental and malformation disorders [6, 8, 9] and have a significant role both in the genetics of complex traits such as autism [10] and in genome evolution [11, 12].

**Figure 1:**
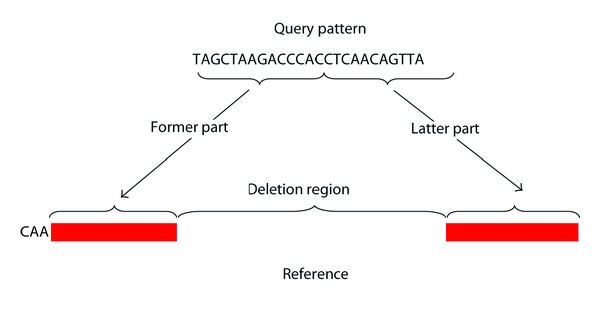
A demonstrative example of large deletion.

In the Smith-Waterman algorithm, to find the optimal local alignment between a query and a reference sequence, a scoring system including a set of specified gap penalties is used. However, when a sequence subjected to a large deletion is used as a query sequence, its alignment with the reference is problematic because the query sequence must be first divided into two parts: the one before the deletion that we will refer to as the “former part” and the one after the deletion that we will call the “latter part” of the query (Figure 1). Otherwise, the classical SW algorithm encounters with the large deletion, and then it will start using gaps. As each gap used contributes penalty points to the alignment score, SW cannot bridge these two parts to be aligned with far apart loci in the reference sequence, and, for example, it can align not only the former part of the query but also an intermix of gaps and some nucleotides from the latter part that match the deleted fragment by chance. Therefore, the basic Smith-Waterman algorithm has a big error in the estimated deletion position; we will refer to this error as EDPE (Estimated Deletion Position Error), which is measured in number of bases (bp). 

The paper is organized as follows. The classical SW method is explained in Section 2 along with an example demonstrating that it fails when query sequences have large deletions. In Section 3, with a small modification SW is used in an incremental way (called IncrementalPartialAlign). Then, in Section 4, to reduce its long runtime for completing many repetitions of SW alignment runs, we propose a better partial sequence alignment method that we called BinaryPartialAlign. In Section 5, we present our experimental simulations on mtDNA fragments and we conclude in Section 6.

## 2. The Smith-Waterman Alignment Algorithm

The Smith-Waterman (SW) algorithm [3] is an algorithm for performing local sequence alignment, considering segments of all possible lengths to optimize the similarity measure (score). A typical use of SW needs a query sequence as input to be searched within a longer reference sequence, for example, the mithecondrial (mtDNA) reference [13]. To identify where the best match/alignment takes place, SW utilizes a scoring matrix to assign a score to the corresponding nucleotide pairs of the query and the relevant fragment of the reference (it also includes gap penalties). After finding the best alignment (possibly with gaps), it gives the similarity ratio as the fraction (percentage) of bases in the query sequence that were matched with the reference.

Let *l*_former_ and *l*_latter_ denote the lengths of the former and the latter parts of the query sequence. Also let [*a*, *b*] denote the fragment from base position *a* to the base position *b* in the reference that best aligns with the former part of the query (of course, *a* < *b*). Similarly, let [*c*, *d*] denote the fragment from base position *c* to the base position *d* in the reference that best aligns with the latter part of the query (*c* < *d*). For the sake of simplicity, let us assume *b* < *c* (and in fact *b* ≪ *c* for the large deletions). Then, it follows that [*b* + 1, *c* − 1] is the part of the sequence subjected to the deletion (and thus not visible in the query sequence). The length of the deletion can be defined as

(1) $$l_{\text{deletion}}=\left|\left[b+1,\;c-1\right]\right|\;=\;c-b-1.$$

**Figure 2:**
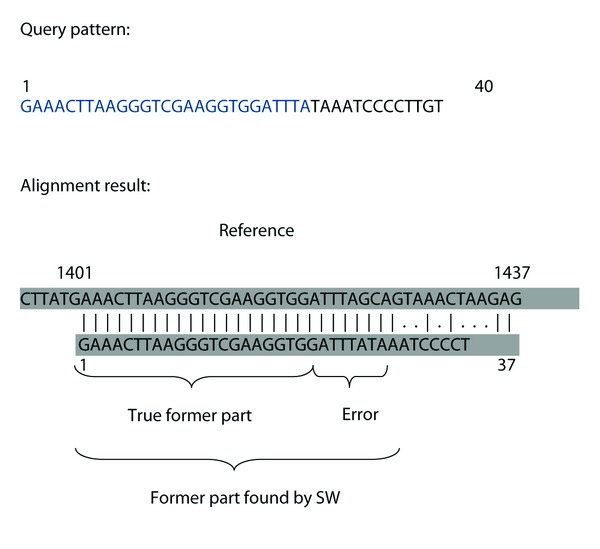
Failure of the classical Smith-Waterman alignment in detecting the large deletion at base position 27. In this simulated experiment, the former part is actually known to be the first 27 nucleotides printed in blue in the “Query pattern”. In “Alignment result”, bases shown in red do actually belong to the latter part but were aligned together with the nucleotides of the former part of the query. SW gives the deletion position estimate of 37, which results in an EDPE of 10 bp.

In Figure 2, we give an example with 40 bp query pattern with a large deletion at base position 27 (i.e., some large fragment was actually removed from between what now appears to be the 27th and the 28th nucleotides). That is, the first 27 bp of the query pattern (the former part) relates to the fragment of the reference from 1401st to 1427th nucleotides, and its last 13 bp (the latter part of the query) is from 2071st to 2083rd nucleotides of the reference sequence. This implies that there was a deletion of length 2071 − 1427 − 1 = 643 (1).

We aligned the query pattern with basic SW to find these two fragments and the location of the large deletion. We used BLOSUM 62 [14] as the scoring matrix, the open gap penalty of 10, the extend gap penalty of 0.5. As shown in Figure 2, the classical SW could not find the large deletion. Instead of 27, it returns 37 bases mapped in the optimal local alignment. This shows that SW has a large estimated deletion position error (EDPE) of 10 bp.

## 3. The Incremental Partial Align Algorithm

To alleviate this problem in a most basic manner, we used SW in an incremental approach and we named this method “IncrementalPartialAlign”. This method takes four input parameters, query sequence, reference sequence, similarity ratio threshold, and minimum sequence length. After aligning the query, if the similarity ratio is found to be below the given threshold, considering that this can be due to the presence of a large gap (small gaps would be tolerated by the classical SW anyway), we try splitting the query into a former and a latter part in all possible ways. We start with the shortest possible former part, the length of which is determined by the minimum sequence length. For example, if the minimum sequence length parameter is 20, we take the first 20 bases of the query pattern. Starting with such a short former part results in a high similarity ratio, but then, we repeatedly increase its length (one by one) until the similarity ratio becomes smaller than the threshold value, at which position we assume that the deletion starts. Then we align the remaining (latter) part of the query pattern with the reference (see Algorithm 1).

**Figure 3:**
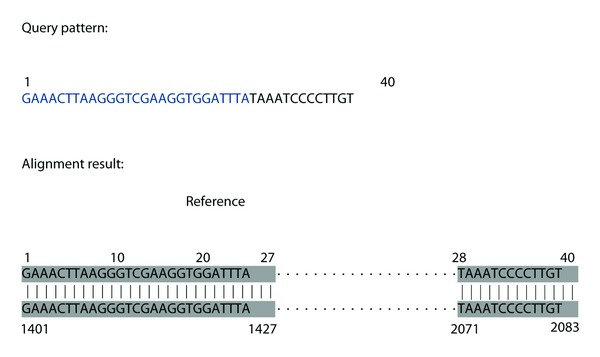
The large deletion at base position 27 is correctly detected by both the IncrementalPartialAlign method and the BinaryPartialAlign method.

The query pattern used in Figure 2, for which the classical SW failed, was also aligned using this IncrementalPartialAlign method (see Figure 3). IncrementalPartialAlign found the deletion at the 27th base correctly (EDPE = 0). The former part is correctly mapped to the 1401st and 1427th, and the latter part is correctly mapped to the 2071st and 2083rd base positions of the reference sequence. However, even though the query pattern was very short (designated for a demonstration only), IncrementalPartialAlign takes 187 msecs for the extensively repetitive use of SW. For longer query patterns (especially when the deletion is present towards the end of the query pattern), alignment would take much longer as many more iterations of SW are used. We propose to modify it using a binary search-based approach [15] that we named BinaryPartialAlign. 

## 4. Proposed Partial Alignment Method Based on Binary Search

Having seen that the classical SW fails in finding large deletions and IncrementalPartialAlign takes more iterations and long time for completing many repetitions of SW runs, we developed a partial sequence alignment method that we called BinaryPartialAlign. The BinaryPartialAlign method uses the same set of four input parameters (query sequence, reference sequence, similarity ratio threshold, and minimum sequence length) as the IncrementalPartialAlign method does. Again, similarly, BinaryPartialAlign is also based on repetitive use of SW. However, unlike IncrementalPartialAlign, it shifts the boundary between the former and latter parts by more than one base in every iteration based on an approach that resembles binary search.

**Figure 4:**
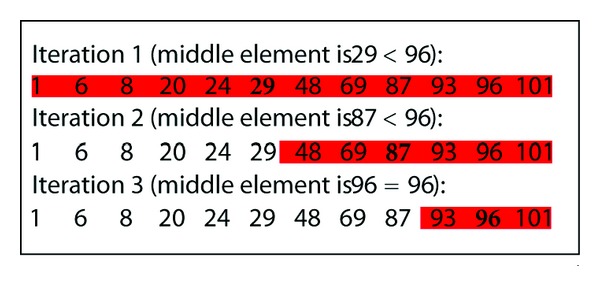
Binary search demonstration. The searched item is 96. The binary search finds the item in 3 comparisons; however, a sequential search would take 11 comparisons.

The binary search is a fast way to search a key within a sorted array. Firstly we check the element in the middle. If the key is equal to that, the search is finished. If the key is less than the middle element, we perform a binary search for the key within the first half. Otherwise (if it is greater), we perform a binary search within the second half. This procedure is demonstrated for searching 96 in a sample integer sequence in Figure 4. Binary search is an efficient search algorithm with a time complexity of O(log⁡_2_⁡*n*). Given *n* elements to search within, the binary search method takes at most log⁡_2_⁡*n* iterations (i.e., makes log⁡_2_⁡*n* comparisons with the key in the worst case, with each comparison reducing the search space to its half).

After aligning the query, if the similarity ratio is above the given threshold, similar to IncrementalPartialAlign, we conclude that there is no deletion in this query and terminate the algorithm. Otherwise, in contrast to the IncrementalPartialAlign, we divide the query pattern into two equal length parts: first half is called the former part and the second half is called the latter part of the query as before. In other words, let *l*_query_ be the total length of the query; we start with a former part and a latter part of length *l*_query_/2.

Then, we align both parts separately and get two similarity ratios to compare with the given similarity ratio threshold. If both of them are above the threshold, we assume that deletion position is found and terminate the algorithm with the current former and latter parts as the output.

If only one of the similarity ratios is greater than the threshold, we lengthen the part with the bigger similarity ratio (and thus shorten the other one) by

(2) $$k=\frac{l_{\text{query}}}{2^{i+\;1}},$$

where *i* is the number of the current iteration (the algorithm starts *i* with an initial value of 1 and increments it until both ratios go above the threshold; see Algorithm 2).

To demonstrate the alignment procedure of BinaryPartialAlign, we used the same query pattern as in demonstration of the classical SW and IncrementalPartialAlign methods. BinaryPartialAlign correctly found the deletion at position 27 with an EDPE of 0 (see Figure 3).

The steps taken by the algorithm can be outlined as follows. Firstly, the query is split into two subsequences with lengths 20 and 20 (former and latter parts, resp.). Then, we increase the length of the former part as its similarity ratio is above the threshold and it is below for the latter part. We obtain 30–10 splits this time. Then, the former part has a similarity ratio below the threshold, which means that the latter part is lengthened. Thus, we obtain 25–15 splits. Then procedure is iterated similarly and stops at 27–13 splits, a perfect partial alignment with EDPE = 0.

When we aligned the query pattern with IncrementalPartialAlign, the alignment time was 187 msecs, which improves to 109 msecs with BinaryPartialAlign. If the query pattern is long and a large deletion occurred towards the end of the query, the alignment would take much more time with IncrementalPartialAlign; however, the BinaryPartialAlign method is not badly affected by neither the position of deletion or the length of the query as it is an O(log⁡_2_⁡*n*) algorithm.

## 5. Simulation Results

In order to test and compare these three alignment techniques, the classical SW, IncrementalPartialAlign, and BinaryPartialAlign were implemented in Java language using the JAligner package [16]. These executables are made publicly available on the site http://ce.istanbul.edu.tr/bioinformatics/PartialAlignment/.

We performed our simulations using 900 samples obtained from the mtDNA reference sequence [13]. Two subsequences selected from the mtDNA reference sequence were concatenated to form a single query pattern. The lengths and positions of these mtDNA subsequences were selected randomly. This way, we created query patterns with lengths varying in the range of 100 to 999 bases. Specifically, to evaluate the effect of the query pattern length, we created 10 length intervals: 100–199, 200–299,…, and 900–999. We formed 100 query patterns from each length interval.

We aligned these 900 sequences with the classical SW, IncrementalPartialAlign, and BinaryPartialAlign. For each length interval, we calculated the average and standard deviation of alignment times and EDPE (estimated deletion position error), which are shown in Table 1. We obtained the runtimes of these three methods using single thread on an Intel Core 2 Duo CPU with 2.50 GHz clock and 3 GB RAM. As clearly seen in Table 1, the BinaryPartialAlign method gives the lowest EDPE within shorter runtime than IncrementalPartialAlign. The classical SW is the fastest one but with a very high EDPE.

## 6. Conclusions 

Sequence database searching is among the most important and challenging tasks in bioinformatics. One special case of sequence searching is when large deletions are present in the query fragments, which is called partial or large-gap alignment. Partial alignment has become a need in bioinformatics as they are associated with developmental and malformation disorders, emergence of complex genetic traits, and genome evolution. Although the best choice of sequence search algorithm is Smith-Waterman's, if a sequence is subjected to a large deletion, to find the deletion location with the classical Smith-Waterman (SW) algorithm is inconclusive. In order to handle this problem, one basic approach is to use SW for various splits of the query pattern repeatedly in a systematic, incremental way. This approach that we called the IncrementalPartialAlign method works better, but, for longer query patterns (especially when the deletion is present towards the end of the query pattern), the procedure would take much longer time as it has many more iterations of SW that are used. Therefore, we proposed the BinaryPartialAlign method based on the binary search idea. 

Considering the (partial) alignment time and error in estimated deletion position, the BinaryPartialAlign method gives better results than both IncrementalPartialAlign and the classical SW. Despite the runtime of the IncrementalPartialAlign method is badly affected by the query length and where the deletion occurs within the query, being an O(log⁡_2_⁡*n*) algorithm, the proposed BinaryPartialAlign is more robust to these factors. 

The executable application, a short user-manual, and the query sequences used for simulations are made publicly available on the site http://ce.istanbul.edu.tr/bioinformatics/PartialAlignment/. These tools will be beneficial to researchers for finding large deletions in investigating their role in the genetics of complex traits and in genome evolution.

## Figures and Tables

**Algorithm 1:**
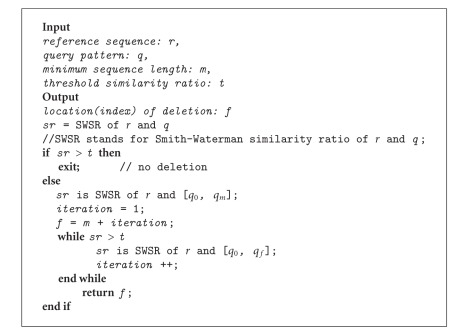
IncrementalPartialAlign pseudocode.

**Algorithm 2:**
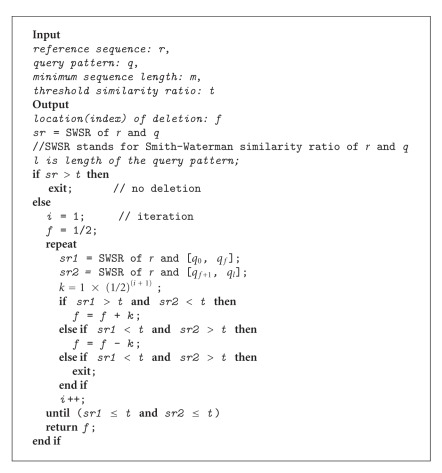
BinaryPartialAlign pseudocode.

**Table 1:** Alignment times of BinaryPartialAlign, IncrementalPartialAlign, and classical SW.

	Alignment time			Base error in estimated deletion position		
	(mean ± standard deviation in msec)			(mean ± standard deviation in bp)		
Sequence length	BinaryPartialAlign	IncrementalPartialAlign	Classical SW	BinaryPartialAlign	IncrementalPartialAlign	Classical SW
100–199	226.7 ± 95.2	3149.0 ± 2460.3	106.6 ± 22.4	9.8 ± 12.3	12.9 ± 11.1	38.6 ± 40.6
200–299	418.3 ± 148.0	6896.9 ± 5484.0	176.1 ± 21.6	13.1 ± 12.3	23.7 ± 20.3	67.1 ± 73.0
300–399	611.0 ± 203.1	16295.1 ± 13346.4	241.4 ± 21.1	21.8 ± 22.8	37.1 ± 30.2	76.0 ± 96.8
400–499	741.6 ± 237.1	28763.0 ± 20988.4	247.0 ± 63.4	29.3 ± 27.9	51.9 ± 33.4	85.9 ± 95.8
500–599	798.8 ± 259.6	38638.7 ± 29724.3	284.3 ± 85.2	37.4 ± 38.9	69.4 ± 50.3	92.2 ± 99.5
600–699	932.9 ± 327.4	60640.8 ± 44106.5	321.3 ± 106.3	41.5 ± 41.1	83.2 ± 53.2	107.6 ± 123.9
700–799	1177.9 ± 413.2	71953.8 ± 59874.8	359.1 ± 128.7	46.2 ± 35.7	94.0 ± 59.8	125.4 ± 167.5
800–899	1241.5 ± 432.4	81953.1 ± 64055.2	395.1 ± 148.0	51.6 ± 46.7	102.9 ± 59	143.7 ± 205.3
900–999	1366.1 ± 482.5	117779.0 ± 84950.1	679.1 ± 33.6	50.9 ± 52.1	127.2 ± 72.5	242.4 ± 285.8

## References

[1] Mount DM (2004). *Bioinformatics: Sequence and Genome Analysis*.

[2] Margulies M, Egholm M, Altman WE (2005). Genome sequencing in microfabricated high-density picolitre reactors. *Nature*.

[3] Smith TF, Waterman MS (1981). Identification of common molecular subsequences. *Journal of Molecular Biology*.

[4] Rognes T (2011). Faster Smith-Waterman database searches with inter-sequence SIMD parallelisation. *BMC Bioinformatics*.

[5] Muratet MA (2002). Comparing the speed and accuracy of the Smith and Waterman algorithm as implemented by MPSRCH with the BLAST and FASTA heuristics for sequence similarity searching. *The Scientific World Journal*.

[6] Conrad DF, Andrews TD, Carter NP, Hurles ME, Pritchard JK (2006). A high-resolution survey of deletion polymorphism in the human genome. *Nature Genetics*.

[7] Ye K, Schulz MH, Long Q, Apweiler R, Ning Z (2009). Pindel: a pattern growth approach to detect break points of large deletions and medium sized insertions from paired-end short reads. *Bioinformatics*.

[8] Schmickel RD (1986). Contiguous gene syndromes: a component of recognizable syndromes. *Journal of Pediatrics*.

[9] Gardner RJ, Sutherland GR (2004). *Chromosomes Abnormalities and Genetic Counseling*.

[10] Yu CE, Dawson G, Munson J (2002). Presence of large deletions in kindreds with autism. *American Journal of Human Genetics*.

[11] Petrov DA (2002). Mutational equilibrium model of genome size evolution. *Theoretical Population Biology*.

[12] Olson MV (1999). When less is more: gene loss as an engine of evolutionary change. *American Journal of Human Genetics*.

[13] http://www.ncbi.nlm.nih.gov/pubmed/7219534?dopt=Citation.

[14] http://www.ncbi.nlm.nih.gov/Class/FieldGuide/BLOSUM62.txt.

[15] Cormen TH, Leiserson CE, Rivest RL, Stein C (2001). *Introduction to Algorithms*.

[16] http://jaligner.sourceforge.net/.

